# Supplementary material for: Tuning phononic and electronic contributions of thermoelectric in defected S-shape graphene nanoribbons
Source: Sci Rep. 2022 Nov 1;12:18419. doi: 10.1038/s41598-022-22379-7 (PMC9626499; doi:10.1038/s41598-022-22379-7)
Supplement: Supplementary file 1 — Supplementary Information. [file 41598_2022_22379_MOESM1_ESM.docx]

Supplementary information:

**Tuning Phononic and Electronic Contributions of Thermoelectric in defected S-Shape Graphene Nanoribbons**

M.Amir Bazrafshan, Farhad Khoeini*

*Department of Physics, University of Zanjan, P.O. Box 45195-313, Zanjan, Iran*

**Corresponding author’s email: khoeini@znu.ac.ir*

Here, the formalism of the NEGF is given in detail, although this method is presented in the literature, but it is in agreement with the notation of this work. The figures regarding a device length $L_{D}\approx47.96$ are presented here.

The retarded Green’s function reads [1]:

| $G\left( E \right)=\left[ \left( E+i\eta\right)S_{D}-H_{D}-\Sigma_{LC}\left( E \right)-\Sigma_{RC}\left( E \right) \right]^{-1}$, | (1) |
| --- | --- |

where $E$ is the energy, $\eta$ is an arbitrarily small positive number, and $\Sigma_{LC(RC)}$ is the self-energy for the left and right contacts, which are given by  [2]:

| $\Sigma_{LC}=\left( \left( E+i\eta\right)S_{D-LC}-H_{D-LC} \right)G_{0,0}^{LC}\left( E \right)\left( \left( E+i\eta\right)S_{LC-D}-H_{LC-D} \right)$,  $\Sigma_{RC}$= $\left( \left( E+i\eta\right)S_{D-RC}-H_{D-RC} \right)G_{2,2}^{RC}\left( E \right)\left( \left( E+i\eta\right)S_{RC-D}-H_{RC-D} \right)$, | (2a)  (2b) |
| --- | --- |

where $G_{LC\left( RC \right)}\left( E \right)$ is the isolated left (right) contact surface Green’s function. Retarded surface Green’s functions are also given by:

| $G_{0,0}^{LC}\left( E \right)=\left( \left( E+i\eta\right)S_{0,0}^{LC}-H_{0,0}^{LC}-(\left( E+i\eta\right)S_{-1,0}^{LC}-H_{-1,0}^{LC})\tilde{\Lambda} \right)^{-1},$  $G_{2,2}^{RC}\left( E \right)=\left( \left( E+i\eta\right)S_{2,2}^{RC}-H_{2,2}^{RC}-(\left( E+i\eta\right)S_{2,3}^{RC}-H_{2,3}^{RC})\Lambda\right)^{-1},$ | (3a)   (3b) |  |
| --- | --- | --- |

where $\Lambda$ and $\tilde{\Lambda}$ are transfer matrices. Transfer matrices for the contacts can be obtained as:

| $\tilde{\Lambda}=\tilde{t}_{0}+t_{0}\tilde{t}_{1}+t_{0}t_{1}\tilde{t}_{2}+\cdots\cdots\cdots+t_{0}t_{1}t_{2}\cdots\tilde{t}_{n} ;$  $\Lambda=t_{0}+\tilde{t}_{0}t_{1}+\tilde{t}_{0}\tilde{t}_{1}t_{2}+\cdots\cdots\cdots+\tilde{t}_{0}\tilde{t}_{1}\tilde{t}_{2}\cdots t_{n} ,$ | (4a)   (4b) |
| --- | --- |

where$t_{0}$, $\tilde{t}_{0}$, $t_{i}$ and $\tilde{t}_{i}$ for the left contact can be given as:

| $t_{0}=\left( \left( E+i\eta\right)S_{0,0}^{LC}-H_{0,0}^{LC} \right)^{-1}\left( \left( E+i\eta\right)S_{0,-1}^{LC}-H_{0,-1}^{LC} \right) ,$  $\tilde{t}_{0}=\left( \left( E+i\eta\right)S_{0,0}^{LC}-H_{0,0}^{LC} \right)^{-1}\left( \left( E+i\eta\right)S_{-1,0}^{LC}-H_{-1,0}^{LC} \right) ,$  $t_{i}=\left( I-t_{i-1}\tilde{t}_{i-1}-\tilde{t}_{i-1}t_{i-1} \right)^{-1}t_{i-1}^{2} ,$  $\tilde{t}_{i}=\left( I-t_{i-1}\tilde{t}_{i-1}-\tilde{t}_{i-1}t_{i-1} \right)^{-1}\tilde{t}_{i-1}^{2} ,$ | (5a) (5b)  (5c)  (5d) |
| --- | --- |

with I as identity matrix. With a same procedure, one can form the transfer matrices for the right contact. By considering $S_{D}=I$ and other overlap matrices are zero, and also replacing $E$ by $\omega^{2}$ this formalism can be used to predict phonon transport [3]. Since every atom has 3 spatial degrees of freedom, every element of the Hamiltonian matrices, is a $3\times3$ block matrix in the dynamical matrix.

The spectral density operator is given by:

| $\Gamma_{LC\left( RC \right)}\left( E \right)=i\left[ \Sigma_{LC\left( RC \right)}\left( E \right)-\Sigma_{LC\left( RC \right)}\left( E \right)^{\dagger} \right].$ |  |
| --- | --- |

The transmission probability for the electron (phonon) can be evaluated by:

| $T_{e\left( \mathrm{ph} \right)}\left( E \right)=Trace\left[ \Gamma_{LC}\left( E \right)G\left( E \right)\Gamma_{RC}\left( E \right)G\left( E \right)^{\dagger} \right].$ |  |
| --- | --- |

Furthermore, the DOS and local DOS can be obtained using:

| $\mathrm{DOS}\left( E \right)=-\frac{1}{\pi}\mathrm{Im}\left( \mathrm{Tr}\left( G\left( E \right) \right) \right),$ |  |
| --- | --- |
| ${\mathrm{LDOS}\left( E \right)}_{j}=-\frac{1}{\pi}\mathrm{Im}\left( {G\left( E \right)}_{j,j} \right)$. |  |

For phonons, DOS and LDOS are changed to vDOS and vLDOS. As mentioned earlier, for phonon $E$ is replaced with $\omega^{2}$. For each atom, there are 3 values for vLDOS, in this work we summed these values.

Results for $L_{D}\approx47.96$Å are shown in Figure S 1.

|   (a) |   (d) |
| --- | --- |
|   (b) |   (e) |
|   (c) |   (f) |

Figure S 1. (a) Maximum figure of merit for different SV locations together with the corresponding $\mu$, (b) the ratio of $S^{2}$ and $ZT_{\max}$, and (c) electronic and phononic terms as a function of various SV locations, (d) the ratio of $ZT_{\max}$ and its chemical potential as a function of different DV locations, (e) the ratio of the Seebeck square and maximum of ZT, and (f) the ratio of electronic and phononic terms vs DV locations. The device length is$L_{D}\approx47.96$ Å.

The dependence of the ZT to the chemical potential and the temperature is plotted in the manuscript for SV-18-24. However, here the same plot for the pristine system (Figure S 2) and with a DV, specifically the one that induces significant increase in ZT, DV-6-8-pr (Figure S 3), are also included.

Figure S 2. The ZT as a function of chemical potential and temperature for the perfect system with $L_{D}\approx59.03$ Å.

Figure S 3. The ZT as a function of chemical potential and temperature in the presence of the DV-6-8-pr for the system with $L_{D}\approx59.03$ Å.

In Figure S 4, we show the result presented in figure 6 for a system with $L_{D}\approx47.96 Å$.

Figure S 4. Comparison between the pristine case of all studied structures with the pristine GNRs in the S-Shape structure**.** The unit of length is angstrom.

A series of figures related to the vLDOS and LDOS of systems are presented in the following based on increasing lengths.

|   Figure S 5. The system with $L_{D}\approx34.43$Å.The figure shows that the high vLDOS atoms are almost far from the edges. |
| --- |
|   Figure S 6. The system with $L_{D}\approx34.43$Å.The figure shows that the high LDOS atoms are almost far from center (purple dashed line in figure 1). |
|   Figure S 7. The system with $L_{D}\approx47.96$ Å. The atoms with high vLDOS are not concentrated in the middle of the chiral part. |
|   Figure S 8. The system with $L_{D}\approx47.96$ Å. The atoms that are located in the middle, have higher LDOS values from their neighbors. |
|   Figure S 9. The system with $L_{D}\approx59.03$ Å. The high vLDOS atoms are almost on the edges. |
|   Figure S 10. The system with$L_{D}\approx59.03$ Å. The atoms with high LDOS are appeared in the middle of the structure.  (a)  (b)  Figure S 11. (a) The vLDOS, and (b) LDOS for 12-ZGNR. |
| (a)  (b)  Figure S 12. (a) The vLDOS, and (b) LDOS for 10-AGNR. |
|  |

**References**

[1] Y. Wu and P. A. Childs, *Conductance of Graphene Nanoribbon Junctions and the Tight Binding Model*, Nanoscale Res. Lett. 6, 1 (2011).

[2] M. P. L. Sancho, J. M. L. Sancho, and J. Rubio, *Quick Iterative Scheme for the Calculation of Transfer Matrices: Application to Mo (100)*, J. Phys. F Met. Phys. 14, 1205 (1984).

[3] M. N. Luckyanova, J. Garg, K. Esfarjani, A. Jandl, M. T. Bulsara, A. J. Schmidt, A. J. Minnich, S. Chen, M. S. Dresselhaus, Z. Ren, E. A. Fitzgerald, and G. Chen, *Coherent Phonon Heat Conduction in Superlattices*, Science 338, 936 (2012)
